# Body condition explains migratory performance of a long-distance migrant

**DOI:** 10.1098/rspb.2017.1374

**Published:** 2017-11-01

**Authors:** Sjoerd Duijns, Lawrence J. Niles, Amanda Dey, Yves Aubry, Christian Friis, Stephanie Koch, Alexandra M. Anderson, Paul A. Smith

**Affiliations:** 1 Department of Biology, Carleton University, 1125 Colonel By Drive, Ottawa, Ontario, Canada K1S 5B6; 2 Wildlife Research Division, Environment and Climate Change Canada, 1125 Colonel By Drive, Ottawa, Ontario, Canada K1A 0H3; 3 LJ Niles Associates LLC, 109 Market Lane, Greenwich, NJ 08323, USA; 4 New Jersey Fish and Wildlife, 8747 Ferry Road, Millville, NJ 08332, USA; 5 Canadian Wildlife Service, Environment and Climate Change Canada, 801-1550, avenue d'Estimauville, Quebec, Canada G1J 0C3; 6 Canadian Wildlife Service, Environment and Climate Change Canada, 4905 Dufferin Street, Toronto, Ontario, Canada M3H 5T4; 7 United States Fish and Wildlife Service, 73 Weir Hill Road, Sudbury, MA 01776, USA; 8 Department of Environmental and Life Sciences, Trent University, Peterborough, Ontario, Canada K9J 7B8

**Keywords:** automated telemetry, *Calidris canutus rufa*, departure decision, migratory performance, shorebirds

## Abstract

Body condition (i.e. relative mass after correcting for structural size) affects the behaviour of migrating birds, but how body condition affects migratory performance, timing and fitness is still largely unknown. Here, we studied the effects of relative body condition on individual departure decisions, wind selectivity, flight speed and timing of migration for a long-distance migratory shorebird, the red knot *Calidris canutus rufa.* By using automated VHF telemetry on a continental scale, we studied knots' migratory movements with unprecedented temporal resolution over a 3-year period. Knots with a higher relative body condition left the staging site later than birds in lower condition, yet still arrived earlier to their Arctic breeding grounds compared to knots in lower relative body condition. They accomplished this by selecting more favourable winds at departure, thereby flying faster and making shorter stops *en route*. Individuals with a higher relative body condition in spring migrated south up to a month later than individuals in lower condition, suggesting that individuals in better condition were more likely to have bred successfully. Moreover, individuals with a lower relative body condition in spring had a lower probability of being detected in autumn, suggestive of increased mortality. The pressure to arrive early to the breeding grounds is considered to be an important constraint of migratory behaviour and this study highlights the important influence of body condition on migratory decisions, performance and potentially fitness of migrant birds.

## 1. Introduction

Migratory birds have to make decisions about when to leave their wintering grounds and when to arrive at their breeding grounds [1]. Appropriate timing of these events is considered a key element of migratory behaviour (e.g. [2,3]). Early arrival on the breeding grounds allows for an earlier onset of breeding, and thus a longer breeding season [4], higher reproductive success [5] and an increase in reproductive performance [6]. For territorial species, early arrival also can be advantageous in competing for the best breeding territories [7]. However migrating too early can entail considerable costs, even mortality, when birds encounter adverse weather, poor food supply or potential nesting sites still covered in snow (e.g. [8,9]).

During spring migration, many long-distance migrants employ a ‘time minimization’ strategy, where large fat reserves are used to fuel long-distance flights with minimal stops *en route* [1,10]. Thus, body condition (i.e. size-corrected mass, an indication of fuel reserves) acts as a key constraint for migratory behaviour. Individuals that experience favourable conditions during winter are known to attain higher relative body condition, which can carry-over through the subsequent migration and breeding season [11–13]. Fat reserves available at the end of the non-breeding period can influence the date of departure for spring migration [14–16], as well as the performance of individuals during this migration. Early departing individuals with high fat loads can experience a higher survival probability [17], increased wind selectivity [18] and faster short-distance flights [19]. If individuals arrive to the breeding grounds early or in higher relative condition, an increased migratory performance can ultimately increase reproductive success (e.g. [7,20,21]).

Migration behaviour or performance is not related only to individual quality; weather, and especially wind, plays an important role in the timing, speed and energetic costs of migration. Adverse weather conditions can significantly alter departure and arrival timing [22,23], leading to an increased stopover frequency during migration, and can increase the total migration time [24,25]. Greater fuel reserves (i.e. higher body condition) can allow for increased wind selectivity, because individuals spend more time above a threshold departure mass [18], and therefore have a longer period of time within which to select supportive winds.

For many migratory species, spring migration is rapid. Moreover, because of the strong selection for early spring arrival and the penalties for birds that arrive outside a narrow time window, theory predicts that the inter-individual variation in timing of spring migration should be small [26]. Although body mass and flight style have been linked to migration performance at the interspecific level [27] and intra-specific studies have shown the effect of body condition and the departure direction [28], the relationships between individual body condition, wind, migration performance and fitness have rarely been simultaneously explored. Understanding small inter-individual variation in timing and speed of migration requires data with fine temporal resolution. Until recently, tracking technologies for small-bodied migrant birds have not offered both large-spatial coverage for tracking long-distance migrations and fine temporal resolution for understanding behaviour.

We used automated VHF telemetry on a continental scale (e.g. [29]) to track the migration of red knots *Calidris canutus rufa* as they travelled from the Atlantic Coast of the USA to the Canadian subarctic and back. In the spring of 2014–2016, we tagged 302 individuals at Delaware Bay, USA: the most important staging site in the Atlantic Flyway for spring migrants heading to their breeding grounds. We tracked a sample of these individuals as far north as James Bay (1500 km north of Delaware Bay), and some individuals through to the subsequent southbound migration in autumn (electronic supplementary material, figure S1). We investigated the influence of relative body condition (here defined as size and time-corrected relative mass; see Material and methods) at Delaware Bay on individuals' departure decisions, migratory performance and timing of arrival to James Bay at the southern entrance to the breeding grounds to test the hypothesis that individuals with higher relative body condition have improved migratory performance. Specifically, we predict that individuals with a higher relative body condition should be more wind selective when departing the stopover site [30], migrate faster and arrive earlier on the breeding grounds [31]. If body condition carries over across seasons [5], individuals with higher relative body condition while at the stopover site could have higher reproductive success [21] and/or improved survival [17]. Because red knots have biparental incubation and brood their young for up to 18–24 days [32], we assume that duration of birds' stay in the Arctic correlates with their reproductive success; here, we relate relative body condition in spring to the timing of southward migration to test the hypothesis that individuals arriving at the breeding grounds earlier and in higher condition have improved reproductive success. We also relate relative body condition at capture in spring to the detection probability in autumn, to test whether individuals with a higher relative body condition during northward migration are more likely to survive to the following season. Our results demonstrate the utility of these fine-temporal scale data for testing predictions of optimal migration theory. Perhaps more importantly, our results underscore the need to maintain suitable feeding conditions for migratory birds at key staging sites, as long-distance migrant birds of various taxa continue to decline around the globe.

## 2. Material and methods

### (a) Background

Red knots are medium-sized shorebirds with a global distribution. The *rufa* subspecies winters primarily in South America and migrates long distances to their breeding grounds at low- and mid-Arctic latitudes in Canada. A large part of the population stages in Delaware Bay (New Jersey and Delaware, USA) during spring migration [33]; it is the single most important spring staging site for red knots before they reach their Arctic breeding areas [34]. While staging at Delaware Bay, northbound red knots feed on the eggs of spawning horseshoe crabs *Limulus polyphemus* [35]. Commercial harvest has reduced the abundance of crabs' eggs and reduced fuelling rates for staging red knots [17,34].

### (b) Capture of birds and tag deployment

Red knots were captured between 16 and 25 May 2014–2016, at several sites around Delaware Bay (39°02′ N–39°42′ N and 74°34′ W–74°94′ W; electronic supplementary material, figure S1). All individuals were measured using standard protocols, and in most cases, bill (±1 mm), flattened wing chord (±1 mm) and mass (±1 g) were measured. Immediately after these measurements were taken, randomly selected birds were fitted with a 1.0 g (approx. 0.7% of body mass), coded radio-transmitter (Lotek Wireless Inc., Newmarket, ON, Canada). These radio tags had a burst interval of 5.9–6.1 s, and an estimated minimum lifespan of 146 days. The radio tags were fitted by clipping a small area of feathers from the synsacral region and gluing the tags to the feather stubble and skin with a cyanoacrylate gel adhesive (Loctite Super Glue Gel Control, Henkel, USA).

The tags were tracked using a network of automated radio telemetry receiving stations, the Motus Wildlife Tracking System, distributed across North America (and to a lesser extent internationally) and concentrated at key shorebird staging sites [29]. Receiver stations consisted of an 8–15 m high tower, equipped with either Lotek SRX600 digital telemetry receivers or a SensorGnome receiver (www.sensorgnome.org). Receiver stations had multiple directional antennas oriented at fixed angles (typically 3–6 nine-element Yagi antennas distributed evenly around 360°) that scanned continuously for tags. All tags operated on a single frequency and were distinguished by a unique series of pulses contained within each burst of radio transmission, allowing for definitive identification of individuals. Birds with tags can be detected at distances of at least 12 km when in flight with direct line of sight to the receiver [36], with numerous examples of detections at much larger distances (up to 75 km; P Loring 2015–2016, unpublished data). Tag detections were recorded by the automated receivers and time stamped by an internal GPS clock. This system allowed us to track individual movements at a continental scale with a temporal resolution of ±6 s.

### (c) Analysis of automated telemetry data

The data were processed following procedures described in [36,37]. In summary, the data recorded for each detection included tag identification (i.e. individual bird), date, time (UTC), coordinates of the receiver station, orientation of the receiving antenna and signal strength (nonlinear scale: 20–255). Owing to the high gain on the receivers, local radio-frequency interference can yield false detections; data were filtered to eliminate false detections by comparing received signals to expected signals on the basis of the unique series of coded pulses that describe the individual tags' identities. In addition, we post-processed detections by determining the precise interval between transmissions on each tag (the burst rate, i.e. 5.9 or 6.1 s) and then removed signals not matching that interval to within ±0.02 s. This filtering ensured that the resulting dataset contained only valid detections of tagged birds.

To distinguish between true (directional) migratory movements and local movements within and among adjacent sites, we grouped all receiver stations within a 50 km radius (electronic supplementary material, figure S1), and calculated a mean latitude and longitude for these regional station groupings (hereafter ‘sites’). Simultaneous or varying detections at towers within these sites were interpreted as stationary or locally moving individuals. Once we identified true migratory movements, individual departure times (UTC) from each site were determined as the last detection per receiver station. Arrival times (UTC) were determined as the first detection in a series of at least three consecutive detections at a receiver station at a different site. Ground speed was calculated as the orthodrome distance (metres) between two or more different receiver stations, divided by the total time (seconds) between departure and arrival. Minimal flight speeds of greater than 5 m s^−1^ were assumed necessary for flight [38], and flight speeds below this threshold were considered as possible stopover or detours *en route* and were excluded from calculations of ground speed. The stopover duration for each individual after it departed the capture site was calculated as the sum of all the time differences (seconds) between the last and first detection per receiver station *en route.* A stopover event was defined as a minimum duration of 30 min, and shorter durations were considered a flyby.

We defined birds as crossing into the breeding grounds when they passed our receiving stations in southern James Bay (51°4′ N, 80°4′ W; electronic supplementary material, figure S1). This area is located at the southern edge of the knots' breeding range and most (81%) of the final detections in spring were recorded at this site. Although there are other receiver stations farther north in the Arctic islands (*n* *=* 4), not all of these stations were operational at the time of arrival of the birds. Many autumn detections were recorded by our receiver stations in eastern Canada. To test whether individuals with a higher relative body condition during northward migration are more likely to survive through to the following season, we used all autumn detections from birds captured in Delaware Bay in spring from any receiver to determine the presence of birds during southward migration. In order to test the hypothesis that individuals arriving to the breeding grounds earlier and in higher condition have improved reproductive success, we only used individuals, that were detected in James Bay during northward migration and consequently were detected at receiver stations around the Bay of Fundy (45°4′ N, 64°3′ W), the Mingan Archipelago (50°1′ N, 63°3′ W) and adjacent sites (less than 100 km) on southbound migration.

### (d) Wind data

We obtained local wind data to relate them to departure decisions and migratory performance. Several past studies suggest that birds time their departures to maximize initial wind assistance (e.g. [39,40]). In addition, because tailwinds encountered *en route* can explain differences in migration duration [41], we obtained the wind speed for each receiver location, for the times at which tagged individuals were present. Wind data were obtained from the NCEP/NCAR Reanalysis project [42], as provided by the NOAA/OAR/ESRL PSD, Boulder, CO, USA, and tailwind estimates were calculated using R package RNCEP [43]. For each individual, we extracted tailwind at departure (3 h interval) and mean tailwind (m s^−1^) of the total trajectory (i.e. integrating wind speeds for all locations and times where birds were detected *en route*). To calculate tailwind, we assumed the airspeed of the red knots to be 16 m s^−1^ [44]. The best tailwind assistance of three different pressure levels was considered (‘surface level’, 700 and 850 hPa), corresponding to altitudes between 0 and 1500 m altitude.

### (e) Data analysis

Mass varies during the stopover, and differs for birds of different structural size. In order to estimate an index of ‘relative body condition’, we used a generalized linear mixed model (GLMM) with mass at capture as the response variable, catching date (day of year) as an explanatory variable, wing length as a covariate and year as random factor. We also considered models including bill length and all possible interactions as predictors. The function dredge in the R-package MuMIn [45] was used to select the most parsimonious model on the basis of AICc [46]. We used the Kenward–Rogers method for approximating degrees of freedom and standard errors [47]. We then used the partial residuals of this analysis as an index of relative body condition in further analyses. Thus, our index of relative body condition reflects the time- and size-corrected relative mass of individuals during their refuelling at the stopover site. A positive value reflects an individual that has a high relative body mass in comparison to the expectation for the population, which we interpret as an individual with an above-average relative fuel load for that day.

We ran separate GLMMs for timing of departure, ground speed, tailwind assistance at departure, average tailwind along the entire migratory route, stopover duration and arrival timing to the subarctic. All models included relative body condition index as a predictor and year as a random factor. For each predicted variable, we started with a full model (including total distance (kilometres) and tailwind at departure, average tailwind and capture date), and then simplified the model using a backward elimination based on a log-likelihood ratio test with *p* < 0.05 as the selection criterion (‘drop1’ in R) until reaching the minimal adequate model. For the analysis of timing of autumn migration (i.e. first detection after the breeding season), we used a linear model to relate date of first autumn detection to fixed effects of relative body condition and year. We determined the detection probability in autumn (yes/no) as a function of relative body condition at capture in spring using logistic regression with year as a fixed factor. Model assumptions were assessed using residual diagnostic plots, and all analyses were performed in the statistical software program R, v. 3.3.3 [48].

## 3. Results

A total of 302 knots were fitted with a transmitter between 2014 and 2016, and we determined accurate departure times for 254 (84%). For the remaining birds, tags may have malfunctioned or fallen off, or the birds may have died prior to departure or escaped detection. Of the 254 individuals for which timing of departure from Delaware Bay was known precisely, 46% (*n* = 117) were detected at least once thereafter and 22% (*n* = 56) were detected at James Bay at the southern edge of their breeding range. After the breeding season, 16% (*n* = 47) of all tagged individuals were detected at various receiver stations.

**Figure 1.**
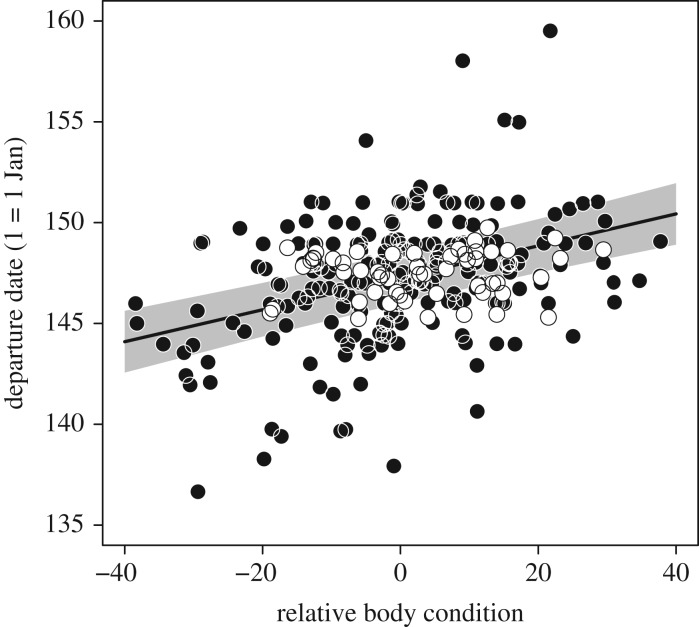
Estimates of the relationship between relative body condition at capture and the timing of departure from Delaware Bay. Individuals with higher relative body condition at capture departed later than individuals with a lower relative body condition. The open dots represent the individuals that were successfully detected entering the Arctic breeding grounds, and the closed dots represent all individuals for which accurate departure times were recorded. Relative body condition (i.e. relative mass after correcting for structural size) is estimated from a linear mixed effects model (see text), and the grey area represents 95% confidence interval for the predicted relationship.

**Figure 2.**
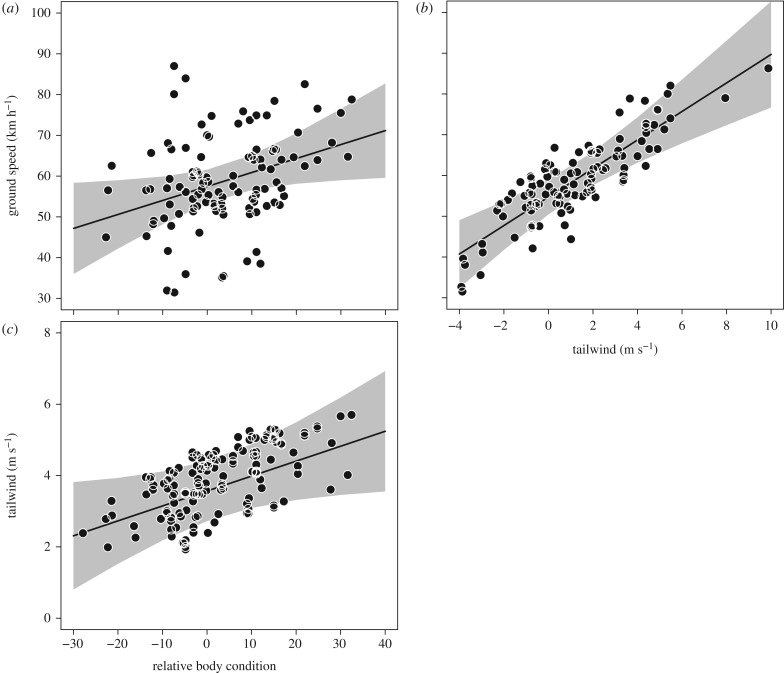
Estimates of the relationship between (*a*) relative body condition and ground speed (km h^−1^), where individuals with a higher relative body condition have higher ground speeds. This appears to be primarily an effect of tailwind at departure, as (*b*) shows the positive relation between tailwind at departure and ground speed and (*c*) shows the relation between relative body condition and mean tailwind along the entire route. Relative body condition (i.e. relative mass after correcting for structural size) is estimated from a linear mixed model (see text), and the grey area represents the 95% confidence interval.

Body mass at capture ranged from 92 to 226 g, and over the narrow interval of capture dates, showed a strong positive relationship with date of capture (GLMM, *χ*^2^ = 103.7, *p* < 0.001). Similarly, there was a negative relationship between the (absolute) body mass at capture and the time spent at the site after capture; individuals captured with a lower body mass remained longer at Delaware Bay before departing for the breeding grounds (GLMM, *χ*^2^ = 43.1, *p* < 0.001). After controlling for effects of date of capture, individuals that were captured with a higher relative body condition departed from the capture site at later dates (GLMM, *χ*^2^ = 6.5, *p* = 0.010; figure 1) and had higher ground speeds (km h^−1^) compared with individuals in lower relative body condition (GLMM, *χ*^2^ = 4.8, *p* = 0.027; figure 2*a*). Wind played an important role in these differences in ground speed, and, as expected, ground speed was positively correlated with tailwind at departure (GLMM, *χ*^2^ = 21.7, *p* < 0.001; figure 2*b*). Birds captured at Delaware Bay with a higher relative body condition appeared to be more wind selective, generally having more profitable winds along the entire route (GLMM, *χ*^2^ = 3.8, *p* = 0.040; figure 2*c*). After departing from their stopover site, knots with a higher relative body condition had shorter stopover durations (GLMM, *χ*^2^ = 6.5, *p* = 0.009).

Arrival date at the southern entrance to the breeding grounds was positively correlated to the departure date (GLMM, *χ*^2^ = 46.9, *p* < 0.001) and negatively related to relative body condition (GLMM, *χ*^2^ = 5.7, *p* = 0.010; for details see electronic supplementary material, appendix S1 and figure S2). Over the range of relative body condition observed in our sample, these relationships suggest that individuals with a higher relative body condition left the final staging site approximately 1.5 days later, achieved ground speeds of 25 km h^−1^ faster, had approximately 1.5 days shorter stopover duration *en route*, and arrived to James Bay approximately 2 days earlier than individuals in lower relative body condition.

**Figure 3.**
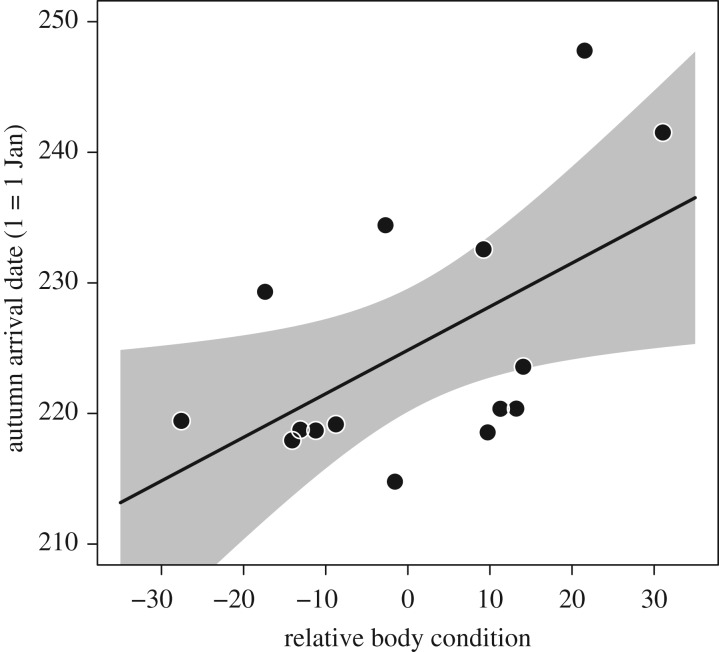
First autumn detection dates at the Bay of Fundy and the Mingan Archipelago in relation to relative body condition at capture during spring migration, for individuals that were successfully detected in both seasons. The positive correlation between relative body condition at capture in spring and the first autumn detection date could suggest that individuals with a high relative body condition at capture had a successful breeding attempt. The grey area represents 95% confidence intervals.

**Figure 4.**
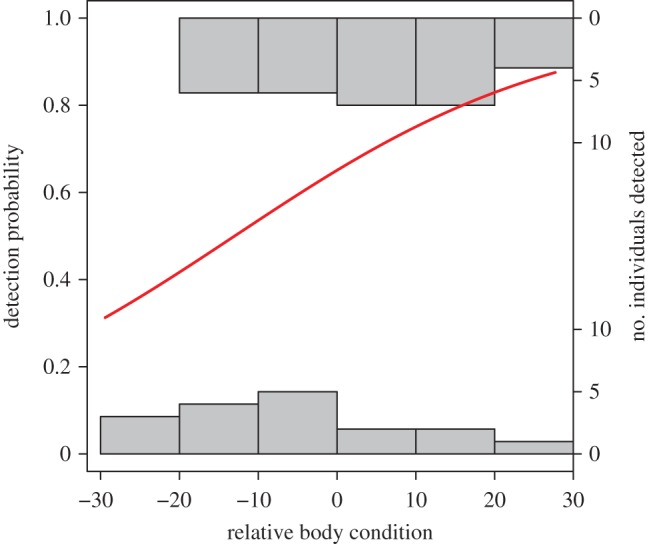
Probability of detection in autumn, and the number of individuals detected, in relation to the relative body condition at capture. The solid line represents the predicted values of the logistic regression in relation to relative body condition at capture. The horizontal bars represent the number of individuals that were not detected (0) and birds that were detected (1). Birds with higher relative body condition at capture in spring were more likely to be detected in autumn. (Online version in colour.)

Of the 56 individuals that were detected entering the Arctic breeding grounds during northward migration, we successfully detected 15 individuals during southward migration. We found a negative correlation between the relative body condition in spring and the date in autumn at which they were first detected at the Bay of Fundy and the Mingan Archipelago (*F*_2,12_ = 3.4, *r*^2^ = 0.36, *p* = 0.029; figure 3), indicating that birds with a higher relative body condition in spring remained longer in the breeding areas; our model predictions suggest a stay of up to 25 days longer over the range of body conditions observed in our sample. We also found that individuals with higher relative body condition in spring had a greater probability of being detected in autumn, versus individuals in lower relative condition (ß_relative body condition_ = 0.048 ± s.e. 0.02, *z* = 2.00, *p* = 0.041, *n* = 47; birds that departed Delaware Bay in spring and were detected anywhere in autumn; figure 4).

## 4. Discussion

Migration and breeding both entail considerable energetic costs [49], and arriving early to the breeding grounds enhances mate and territory acquisition, extra-pair mating opportunities and opportunities for re-nesting [50]. Therefore, body condition and the timing of and performance during migration can carry over to affect breeding season performance, recruitment and population dynamics in general. Our results demonstrate the important role of body condition immediately before the final legs of migration and underscore the importance of feeding conditions at staging sites for long-distance migrants. We show that, as predicted, individuals with higher relative body condition at a spring stopover site select more supportive winds, have higher flight speeds and stop less long *en route*; this enabled northbound red knots departing Delaware Bay with a higher relative body condition to arrive to James Bay earlier, despite that they left Delaware Bay later. We also show that these individuals in higher relative body condition migrated south up to a month later, suggesting a possible link between condition in spring and reproductive success. In addition, we show that individuals with a high relative body condition in spring have a higher probability of being detected in autumn.

Migration can comprise up to 50% of the annual energy budget for birds [49], and the winds encountered during migration can substantially alter these already high-energy costs. To both minimize energy expenditure and maximize speed, birds are predicted to select supportive winds during their migrations [51,52]. Our results suggest that red knots with a higher relative body condition at capture selected more supportive winds along the entire route and consequently flew faster, partly explaining their earlier arrival to the breeding grounds, despite that in some cases, they remained longer at the stopover site in order to depart with these favourable winds. All individuals face the pressure of the narrow time window of suitable arrival dates in the Arctic [53,54]. The tendency for individuals in lower relative body condition to be less choosy about wind conditions at departure could reflect inexperience [55], or potentially could reflect their cognizance of their low fuelling rate [56], which according to optimal migration theory yields a lower optimum departure fuel load for time-minimizing migrants [57].

Red knots with a higher relative body condition stopped for shorter periods *en route*, thereby saving time and minimizing the non-trivial predation risks associated with stopover [57,58]. Conversely, individuals with a lower relative body condition that stop more often face increased costs of search and settling at new stopover sites [57,59], and possibly increased predation risk [58,60].

Among the red knots tracked to James Bay in spring and then detected again in autumn, we found a negative correlation between relative body condition and the date of first detection in the Bay of Fundy and the Mingan Archipelago in autumn. Up to 25 days delay in southward migration that we observed for individuals in higher condition is much larger than expected due to differences in migratory performance. Similarly, these differences in timing do not seem consistent with a difference in condition and timing of migration among the sexes. Female red knots depart the breeding areas within several days after hatching while the males care for the young until fledging [61]. In red knots, the females tend to be somewhat larger than the males, though much overlap exists [62] and intersexual differences in spring condition (after correcting for size, as we did) are not known. We found no systematic differences in wing or bill length for early versus late autumn migrants, suggesting that the patterns observed are not due to differences in timing among the sexes. Moreover, the dates of the early autumn migrants in our sample (late July) are in accordance with observations from the Arctic of the departure of failed breeders (P.A.S. 2000–2017, personal observations). Therefore, we suggest that birds in high condition had a delayed southward migration because they were more likely to attempt breeding, or were more successful in their attempt, than birds in lower condition.

The suggestion of reproductive failure for birds in low condition during spring migration is complemented by our observation of a reduced likelihood of detecting these low condition birds anywhere during autumn migration, potentially due to mortality. Our results are supported by earlier results of Baker *et al*. [17], where years with overall low rates of mass gain at Delaware Bay were associated with reduced recruitment of juveniles in subsequent years (suggestive of reproductive failure) and where banded individuals with lower estimated departure masses from Delaware Bay were less likely to be resighted in subsequent years (suggestive of adult mortality). In addition, the detection probability for birds in high condition is within the observed range for adult survival (indicating no large-scale tag loss) [17,63].

Our reduced detection in autumn of individuals that were in low relative body condition in spring, while suggestive of mortality, could arise from other potential sources of bias. If low condition individuals aborted northward migration, they would be unavailable for detection in autumn. However, no individuals that were recorded departing from Delaware Bay were observed to abort the migration and then head south in spring (where they would have been detected by other receiver stations in the network). Reduced autumn detection could also arise if low condition individuals began body moult early (at which time the tags, glued to the feather stubble, fall off). Harrington *et al*. [64] scored body moult for this population of red knots while they passed through the USA. They found that during autumn passage in late July and early August, 332/346 (96%) were in complete or nearly complete alternate (breeding) body plumage. Only those birds lingering in the USA into September and October achieved basic (non-breeding) plumage [64], and all of our autumn detections occurred before September. Thus, it seems most likely that our reduced detection of low condition birds in the subsequent autumn relates to increased mortality.

In years of especially high food abundance in Delaware Bay, it has been suggested that late-arriving red knots can increase the rates of mass gain to ‘catch up’ with earlier-arriving birds [34], for example, by spending more time per day feeding [65]. However, for the years of our study, we show that birds with lower relative body condition during spring migration maintain this handicap, showing reduced migratory performance, delayed arrival to the breeding grounds and potentially reduced breeding success and even elevated mortality.

The optimal timing of migration is critical for migratory animals, because mistiming their migration relative to peaks in resource abundance at staging or breeding sites can have negative fitness consequences [66,67]. Many migratory species are advancing their timing of breeding as a result of climate change [68], with most temperature increases occurring in Arctic regions [69]. This warming has led to a change in departure phenology in red knots along the East-Atlantic flyway [67], and it is likely that the red knots in our study, along the West-Atlantic flyway, are faced with similar climatic and phenological challenges. Red knots' use of Delaware Bay is timed to coincide with the timing of horseshoe crab spawning, and thus, phenological constraints and foraging constraints are intertwined. As tracking technologies continue to improve, so too will our understanding of the behavioural decisions made by long-distance migrants, and how these decisions influence and are influenced by individuals' energetic status. For the many species of long-distance migrant birds declining around the globe, including the red knot, this improved understanding of migration behaviour could prove critical for conservation.

## Supplementary Material

AICc comparison of the statistical model for arrival in James Bay; Locations of groupings of automated telemetry receivers in North America; Estimates of the relationship between residual mass (relative body condition) and arrival dates to the sub-Arctic

## Data accessibility

Data are available from the Dryad digital repository [70].
