# Supplementary material for: Body condition explains migratory performance of a long-distance migrant
Source: Proc Biol Sci. 2017 Nov 1;284(1866):20171374. doi: 10.1098/rspb.2017.1374 (PMC5698639; doi:10.1098/rspb.2017.1374)

Online Electronic Supplementary Information for:

Body condition explains migratory performance of a long distance migrant

Sjoerd Duijns, Lawrence J. Niles, Amanda Dey, Yves Aubry, Christian Friis, Stephanie Koch, Alexandra M. Anderson & Paul A. Smith

Table S1. AICc comparison of the statistical model for arrival in James Bay. The top 5 models are displayed, with the best model in boldface. Timing of arrival in James Bay is the response variable in all models. Departure refers to the last detection of an individual in Delaware Bay and tailwind refers to the tailwind the first 3h of the trajectory.

Model	Fixed effects	K_b	AICc	Δ AIC_c	AIC_c weight	Log likelihood
**1**	**Residual mass + Departure + Residual mass*Departure**	**6**	**184.2**	**0.00**	**0.371**	**-85.232**
2	Residual mass + Departure + Residual mass*Departure + Tailwind	7	186.6	2.35	0.115	-85.089
3	Departure + Tailwind + Departure*Tailwind	6	187.2	3.00	0.083	-86.730
4	Departure + Tailwind	5	187.5	3.28	0.072	-88.134
5	Residual mass + Departure	5	187.8	3.58	0.062	-88.285

NB. Year is included as a random intercept in all models.

Fig S1. Locations of groupings of automated telemetry receivers in North America (see main text for details). The white dot indicates the capture site of Delaware Bay and the grey dot represents James Bay, located at the southern edge of the breeding grounds. The red dots indicate the fall detection sites of the Mingan Archipelago the Bay of Fundy. Maps created using R 3.3.3 using packages ggplot2, ggmap, raster and RgoogleMaps (image data providers: US Dept. of State Geographer © 2016)

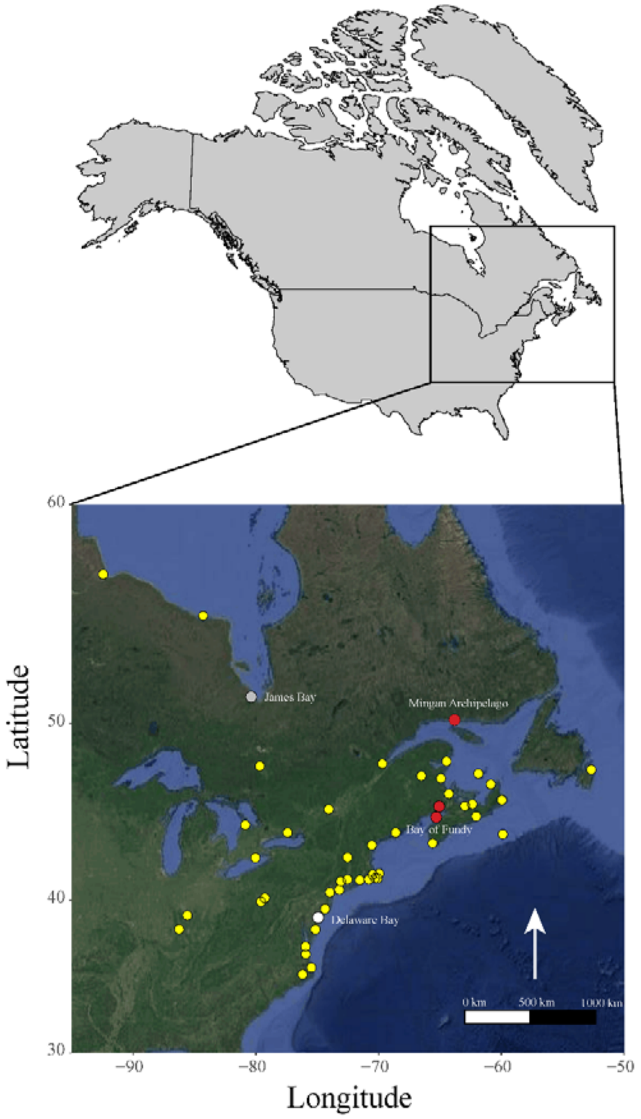

Fig S2. Estimates of the relationship between residual mass (relative body condition) and arrival dates to the sub-Arctic. Birds in a higher condition at the stopover site arrive earlier at the breeding grounds. Data points are estimates of linear mixed models (see main text for details), and the gray area represents 95% confidence intervals.

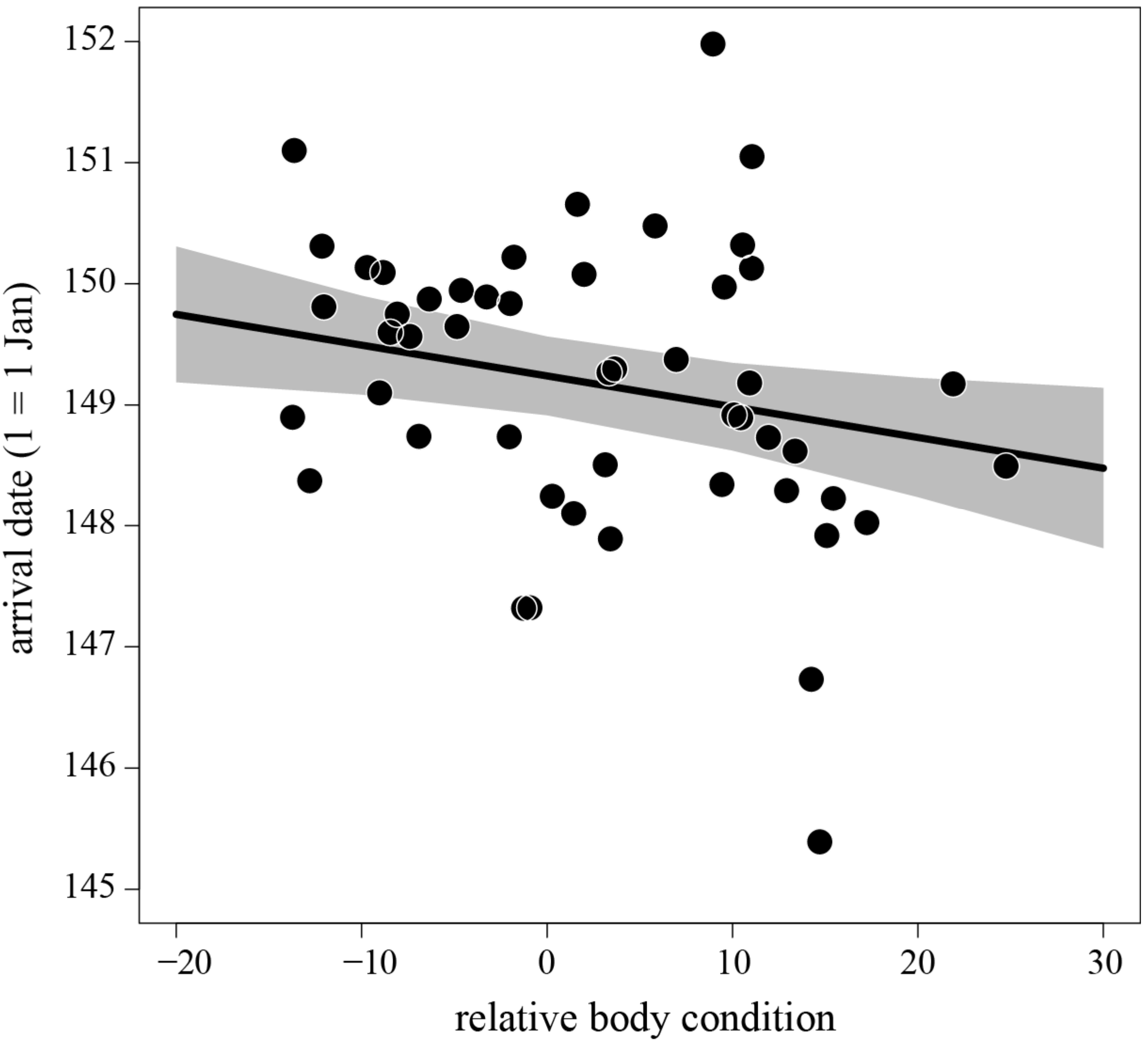

Supplement: AICc comparison of the statistical model for arrival in James Bay; Locations of groupings of automated telemetry receivers in North America; Estimates of the relationship between residual mass (relative body condition) and arrival dates to the sub-Arctic [file rspb20171374supp1.pdf]
